# Global Nursing—a literature review in the field of education and practice

**DOI:** 10.1002/nop2.79

**Published:** 2017-04-09

**Authors:** Mia Kraft, Anne Kästel, Henrik Eriksson, Ann‐Marie Rydholm Hedman

**Affiliations:** ^1^ The Swedish Red Cross University CollegeDepartment of Care and NursingStockholmSweden

**Keywords:** global nursing arena, global nursing competencies, global nursing education, global nursing networking, global nursing practice

## Abstract

**Aim:**

To describe key findings of Global Nursing in empirical nursing studies.

**Design:**

A literature review using descriptive data synthesis of peer‐reviewed articles in the field of nursing education and practice.

**Methods:**

This review of Cinahl Complete, PubMed, PsycINFO and Scopus was undertaken using the search strategy “global nursing”. Independent title/abstract and full‐text screening was undertaken, identifying original articles written in English.

**Results:**

A total of 472 titles and 170 abstracts were read through. Seventy‐three articles were included for full‐text review. Twenty published studies of Global Nursing with multiple research methodologies fulfilled the inclusion criteria. Findings were described with five categories. Global Nursing Arena, Global Nursing Working Environments, Global Nursing Workforce Management, Global Nursing Competencies and Global Nursing Networking were shown to be crucial when Global Nursing was addressed in the literature.

## 1 Background

Globalization is one of the most important driving forces in the new millennium. Together with urbanization and digitalization, it reshapes patterns of human existence and societies on a global scale. Although nursing to some extent has been a global profession, the expansion to global awareness clearly develops the patterns in nursing knowledge. However, there have been few attempts to grasp and overview the consequences of globalization as it is addressed in scholarly knowledge. Researchers stress that nursing in the 21st century has many challenges due to the globalized world (Austin, 2001; Bradbury‐Jones, 2009; Davidson, Meleis, Daly, & Douglas, 2003; Nardi & Gyurko, 2013; Silva, 2004). Furthermore, this global approach in nursing has been reported to correspond to the challenges of human vulnerability and equality. In addition, the strengthening of nursing discipline stands out, both in a local and Global Nursing scene (Bradbury‐Jones, 2009; Davidson et al., 2003).

It has been stressed that the trend towards global justice or fairness as a deeper human rights issue is everyone's responsibility. Benatar, Daar, and Singer (2003) reported that a global agenda could include greater attention to duties, social justice and interdependence. Globalization originally refers to the social and political collaboration of international organizations and people, to improve the quality of civil societies across the world (Crigger, 2008). High mobility, economic interdependence and electronic interconnectedness are positively addressed to globalization. To this date, the rapidly expanding global economy has failed to reduce poverty and improve health for all (cf. Benatar et al., 2003; Bradbury‐Jones, 2009). From one published literature review the findings can be used by policy makers, researchers, clinicians and educators, to guide nursing professionals as Global Nursing provides a framework for better health worldwide (Wilson et al., 2016).

The nursing profession is changing and has gone through several modifications during the last decades (Nichols, Shaffer, & Porter, 2011; Silva, 2004). For that reason, nurses, individually and collectively, have the power and the social consciousness to influence basic social services and health care. Thus, nurses should conceive a broader global picture (Bradbury‐Jones, 2009; Davidson et al., 2003; Silva, 2004). As global competencies must be mastered, a deconstruction of long held values, faculty knowledge, nursing policies, nursing education and research are highlighted as important factors (Bradbury‐Jones, 2009; Crigger, 2008; Nardi & Gyurko, 2013; Parcells & Baernholth, 2014; Riner, 2011).

In an attempt to reconstruct nursing education, a critical reflection on Global Nursing practice is important and how it can be informed by enhanced political awareness. Furthermore, there have been few attempts to conceptualize Global Nursing to address the conclusive parts in the knowledge generated. Therefore, it is of vital importance to describe important aspects of Global Nursing. In addition, the focus in Global Nursing has often been from the perspective of cultural, ethnic and social differences or cultural diversity (cf. Leininger, 1997; Washburn, 1987; Wilson et al., 2016). To overcome this, nurses in clinical practice and nursing students in higher education are challenged to adopt a critical stance to such problematization and conceptualization in nursing. T

he aim of this study was to describe key findings of Global Nursing in empirical nursing studies.

## 2 Methods

A literature review using descriptive data synthesis of previous reported findings was used to conduct this investigation (cf. Evans, 2002; Kmet, Lee, & Cook, 2004).

### 2.1 Sample

An extensive four‐part search strategy was employed to identify relevant studies in the field of nursing education and practice. Research studies focusing on topics concerning Global Nursing were selected. Moreover, review criteria were established by three of the four authors to include articles of primary research or secondary data analysis.

Each phase of the data collection was carried out using six inclusion criteria described in Table 1. Initially, a literature search based on the first inclusion criteria started the process. In accordance with criteria 2–6, the literature search was performed in databases: Cinahl Complete, PubMed, PsycINFO and Scopus. No date restrictions were applied. The use of the database PsycINFO was decided to include an interdisciplinary database with focus on psychology, behaviour and social science related to nursing.

**Table 1:** Inclusion criteria in search strategy and the review

Criterion 1: Language	Literature in English
Criterion 2: Terms/concepts/keywords	Terms/concepts/keywords used (based on the database): Global Nurs*, Global Nursing and the exact term “Global Nursing” were used. Search terms were supposed to appear in title, in title OR in abstract, and both in title and abstract
Criterion 3: Fields of science	Different fields of science concerning the human aspects: Health Sciences (Nursing Science, Medical Science, Physical Education), Social Sciences and Pedagogy.
Criterion 4: Content	Global nursing in educational or professional context.
Criterion 5: Publication	Published empirical studies in valid peer‐reviewed scientific journals Cinahl Complete, PubMed, PsycINFO and Scopus.
Criterion 6: Quality	The quality of the published studies.

In the four databases, studies were excluded if they were critic comments, conference reports, historical discussions, meeting abstracts, opinion pieces or studies focused on globalization issues other than nursing. Moreover, review articles and non‐scientific publications were eliminated and only articles published in peer‐reviewed scientific journals were accepted. Some of the articles were not available with reasonable resources; they were therefore omitted. In addition, disagreements were resolved by consensus between the three authors (cf. Whittemore, 2005; Whittemore & Knafl, 2005).

**Figure 1:**
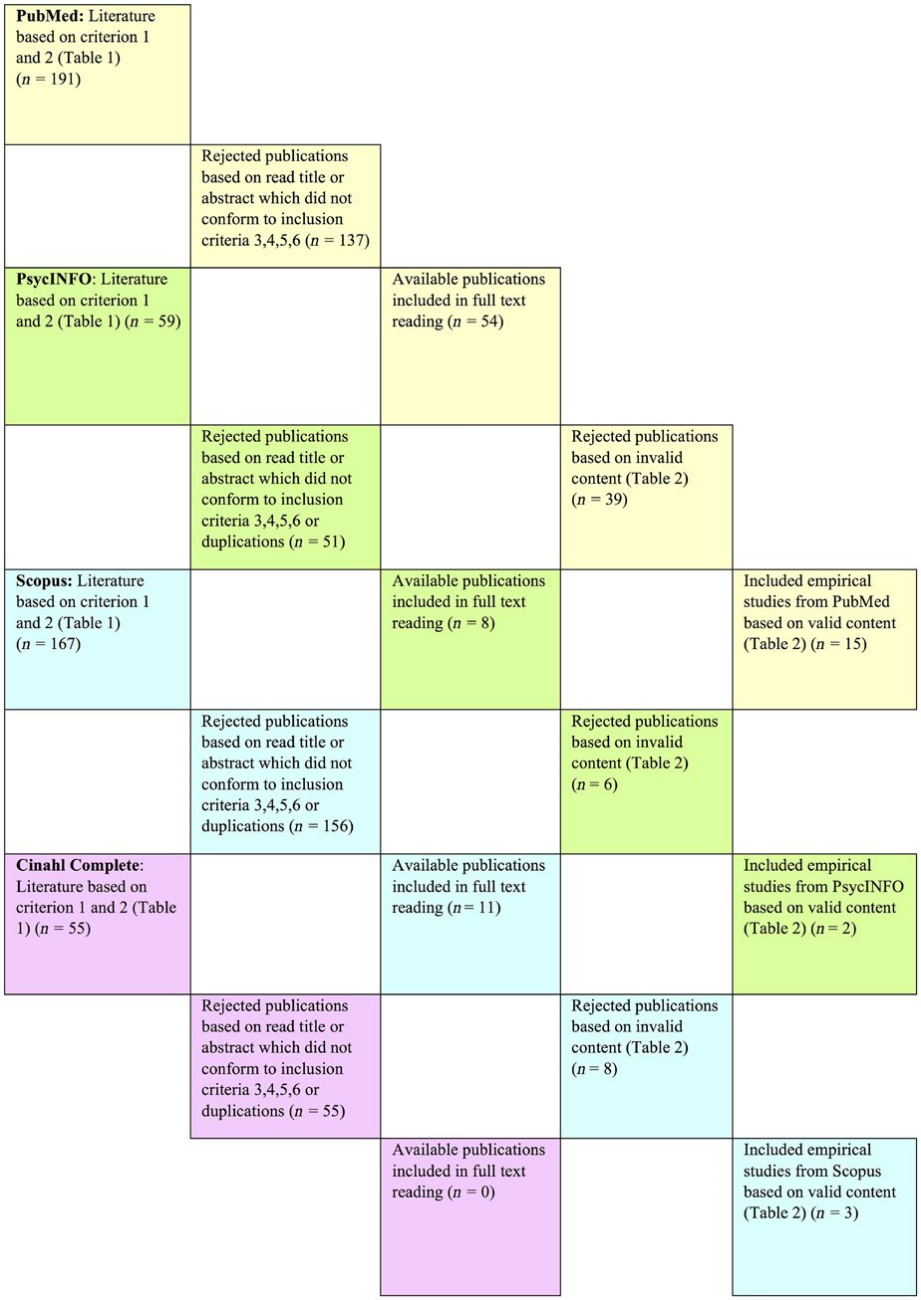
Flow chart of the review process for search and inclusion

A first search in databases was conducted in PubMed using the term “global nurse” or “global nursing” in title/abstract. The progression of the review process is shown in Figure 1. The PubMed search generated 191 articles for potential inclusion. Then, publications were tentatively selected by title. After title and abstract review, 137 were retained. After exclusion, in total, 54 articles were included for full‐text reading (*n *=* *54) after this first search. Next, a second search in databases was applied in PsycINFO using the term “global nurs*”. The PsycINFO search produced 59 articles for potential inclusion. Title review, abstract review and elimination of duplications yielded 13 additional articles from PsycINFO. Eight were included for full‐text reading. Then, a third search in databases was conducted in Scopus using the term “global nurs*” and generated 167 articles for potential inclusion. Title review, abstract review and elimination of duplications yielded 11 additional articles from Scopus for full‐text reading. Consequently, a fourth keyword search in databases was carried out in Cinahl Complete using the term “global nurs*” as a keyword (*n *=* *55). All 55 articles were rejected after title review, abstract review and elimination of duplications.

Finally, after searches in four databases, a total of 472 titles were read through. After reviewing 170 abstracts, 73 articles were included for full‐text review (Figure 1). In the following phase, data collection and article approval were carried out on the basis of the valid content of the full‐text articles.

The appreciation and evaluation of the selected articles (*n *=* *73) were carried out using a checklist described in Table 2. This checklist was created and modified by three of the authors on the basis of available literature. The used checklist (Table 2) was outlined according to Kmet et al. (2004). It was expanded with some components (sections 1–5) and a few components were modified (sections 10) or excluded. Ten specific evaluation sections were included in the checklist and each of these sections consisted of evaluation criteria (Table 2). Studies that accumulated “Yes” in 9 or 10 specific evaluation sections were accepted for the next phase. After the evaluation, 53 articles were rejected based on invalid content. Disagreements were resolved by consensus between three of the authors. Totally, after careful appreciation and evaluation of the full‐text research articles, 20 empirical studies between January 2003 ‐ October 2015 were accepted and included for the findings part of this review (Table 3).

**Table 2:** Checklist with evaluation sections

Evaluation sections	Yes	No
1. Is the type of approach empirical?	The type of approach is empirical study.	There is no description about the type of approach. The type of approach is not empirical.
2. Year of publication	Year of publication is described.	There is no year of publication described.
3. Year of data collection	Year of data collection is described.	There is no year of data collection described.
4. Country for data collection	Country for data collection is described.	There is no country for data collection described.
5. Objective: Is global nursing a part of the reported aim of the study?	There is a clear description of Global Nursing in the objective.	The objective is not mentioned or the objective does not relate to Global Nursing.
6. Context (education or nursing professionals) for the study clear?	A description about the context is clearly defined and suited for the research.	There is no description about the context.
7. Recruitment strategy described, relevant and justified?	The study group is described accurately as is the reason why the group concerned was chosen.	There is hardly enough information about the study group.
8. Data collection methods clearly described and systematic?	Data collection is described accurately.	There is hardly enough information about the data collection.
9. Results and conclusions reported in sufficient detail?	The research questions are described.	There is no description of global nursing in the results part.
10. Ethics described?	The ethical issues of the research are widely stated, inclusive acquisition of anonymity, research approval and contraction of informed consent.	There is no mention or insufficient mention of ethical issues or weaknesses.

**Table 3:** Overview of the included studies

Authors, year, journal, country for publication	Scientific field of the Journal/Aim of the study	Region of data collection, year	Analysis methods	Sampling/data collection	Setting	Participants
Brunetto et al. (2012). *Nursing Outlook*, USA	Nursing science/To investigate a specific component of the quality of management; The supervisor–nurse relationship, supervisor communication, it examines this relationship for its impact on the different generations of nurses.	Australia [Victoria, New South Wales, Western A. Queensland,] 2009	Descriptive statistics, multivariate analysis of variance, path analysis with ordinary least square	Generational cohort/survey	9 urban and regional or metropolitan, medium and smaller hospitals [7 private and 2 public sector hospitals]	900 nurses [post‐ or undergraduate, hospital certificate, technical college, high school]
Garner et al. (2009) *Nurse Education in Practice,* UK	Nursing science/To describe application of the ETINL model providing an ongoing forum for students and faculty members.	UK/USA, 2008	Qualitative analysis, experimental analysis	Cohort/focus group, diary, logbook	3 university nursing faculties in a collaborative World Class using web‐based technology	15‐20 advanced level students, BNE and BSN students, RNs, 2‐6 Faculty members
Gerrish and Griffith (2003) *Journal of Advanced Nursing* UK	Nursing science, Social science/To evaluate a programme with reference to its objectives, outcomes and overall success from the perspective of various stakeholders.	UK, 2001‐2002	Instrumental case study, dimensional analysis	Purposive/focus group, individual interview	An adaptation programme for overseas nurses in Acute care hospitals and one Specialist Cancer Hospital	14 managers, 8 faculty members, 10 mentors, 17 RNs educated in China, Philippines, India, sub‐Saharan Africa
Gutierrez et al. (2012) *Journal Of Advanced Nursing,* UK	Nursing science, Social science/To examine the relations between organizational commitment, organizational support, work values, global job satisfaction among faculty.	USA, 2006‐2007	Descriptive statistics, factor correlation, multivariate analysis	A national stratified random/Survey	By the state approved schools of nursing	1453 Nursing faculty members
Harrowing et al. (2010) *International Nursing Review,* UK	Nursing science, Social science/To examine the ethical principles and considerations that guide health research conducted in international settings using the example of a qualitative study Ugandan nurses/midwives.	Sub‐Saharan Africa/Uganda 2006‐2008	Critical incident analysis	Purposive/focus group and individual interview, participant observation	A programme for skills training in knowledge of HIV and AIDS at a tertiary care hospital	25 non‐resident nurses and nurse‐midwives
Havens et al. (2013) *Journal of Nursing Management,* UK	Nursing science, Social science/To describe staff nurse work engagement, predictors by generational cohort, implications for managers.	USA [Pennsylvania] 2012	Descriptive statistics, correlation, regression analysis	Generational cohort/non‐experimental survey	5 rural, private, non‐profit, non‐religious acute care hospitals	747 RNs [diploma in nursing, associate degree. MSc. baccalaureate degree]
Kim et al. (2006) *Advances in Nursing Science,* USA	Nursing science/To identify factors and strategies to develop global nurse leaders, to describe the educational training experiences nurse leaders received for global nursing work, to compare the competencies for global executives/global nurse leaders.	Unidentified countries in all continents, 2005	In‐depth exploration, content analysis	Purposive/structured individual interview	WHO, ICN, International Work of PHD in Nursing, Chief Nursing Office	17 nurse leaders [15 PHD and RN, 2 other academic fields]
Lesia and Roets (2013) *Africa Journal of Nursing Midwifery,* S.Africa	Nursing science and Midwifery/To report on the placement and usage of advanced midwifery practitioners.	South Africa, 2010	Descriptive statistics, quantitative analysis	Purposive/non‐experimental survey	The University of the Free State	69 Advanced midwifery practitioners [MSc, advanced diploma level]
Meum et al. (2013) *International Journal of Medical Informatics,* UK	Medical informatics/To assess the extent, form, and transformation of global nursing classifications (NANDA) in a nursing practice during a period of 5 years.	Norway, 2005‐2008, 2008‐2010	Longitudinal case study, exploratory analysis	Purposive/individual interview, participant observation, document analysis	A psychogeriatric inpatient ward at an university hospital	Unknown number of clinical staff and 19 nurses and social workers
Ortiga (2014) *Social Science & Medicine,* USA	Social science, Medical science, Health science/To examine the experiences of nurse educators working with poor nations that actively deploy and export nursing labour.	Philippines [Manila, Laguna Palawan, Misa‐mis Oriental],2010‐13	Descriptive, qualitative analysis	Purposive/individual interview	15 nursing schools [12 members of private schools association run by families or corporations, 3 public schools]	34 clinical instructors, 14 deans, 10 nursing school administrators
Squires and Juárez (2012) *International Journal of Nursing Studies,* UK	Social science, Midwifery/To study the perspectives of Mexican nurses about their work environments to determine similarities and differences to results from developed world studies.	Mexico [Leon, Oaxaca, Tamaulipas, Mexico C.] 2006, 2008	Two‐phase case study, In‐depth exploration, content analysis	Convenience and snowball/Semi‐structured individual interview	Primary care settings and acute care settings [private and public sector in hospitals and community clinics]	46 nurses [technical degree, BSN degree, advanced level nurse, MSc]
Swenson et al. (2005) *International Nursing Review,* UK	Nursing science, Social science/To describe both the initial and the subsequent impact of the 2001 Global Nursing Partnerships Conference on the key challenges facing the global nursing community.	UK [London] and 13 countries represented at the conference, 2002	Descriptive statistics, quantitative analysis, content analysis	Purposive/survey	The 2001 Global Partnerships Conference and immediate postconference	63 conference members, 41 national nursing associate leaders, 31 government chief nursing officers
Toren et al. (2012) *Journal of Nursing Management,* UK	Nursing science, Social science/To examine the decision‐making and factors influencing nursing students when choosing a workplace.	Israel, 2008	Descriptive statistics, quantitative analysis, content analysis	Convenience/survey, focus group	The University School of Nursing and a University Medical Centre	47 female secular Jewish and male Arab students in their final year of education.
Tourangeau et al. (2014) *Journal of Nursing Management,* UK	Nursing science, Social science/To identify factors affecting Canadian home care nurse intention to remain employed.	Canada, 2011	Exploratory descriptive, qualitative content analysis	Generational cohort/focus group	Urban and rural home care agencies [employed profit/not‐for‐profit]	50 home care nurses [27 RNs and 23 RPNs]
Zhou et al. (2011) *International Journal of Nursing Studies*, UK	Nursing science, Midwifery/To explore social construction of difference and the related intersection of difference and racialization—China educated nurses working in Australia.	Australia;[Brisbane, Adelaide], 2009	Symbolic interactionist exploratory by GT	Purposive and snowball/individual in‐depth interview	Hospital settings and nursing homes	28 RNs educated with BNE in China
Zinsli and Smythe (2009) *Journal of Transcultural Nursing,* USA	Nursing science/To explore the experience of humanitarian disaster and emergency nursing.	New Zealand, 2008	Heideggerian phenomenology, hermeneutics analysis	Purposive/individual interview	International relief/disaster settings; [Red Cross, Emergency, CARE]	7 nurses, 1 primary researcher/nurse
Walton‐Roberts (2012) *Global Networks,* UK	Sociology, Anthropology/To explore how a colonial discourse of caste‐based pollution has given way to sexual pollution under migration.	India [Kerala] 2008	Descriptive statistics, Nvivo qualitative data analysis	Purposive/survey, focus group, interview/no response rate	3 government nursing colleges, 7 private nursing colleges	1169 faculty, students, officials in medical department/recruitment agency
Weng et al. (2015) *Journal of Nursing Management,* UK	Nursing science, Social science/To explore influences of transformational leadership on nurse innovation behaviour and organization.	Taiwan, 2011	Descriptive statistics, multiple regression and factor analysis	Purposive/survey	3 regional hospitals [internal med., surgery, obstetrics, paediatric, emergency/ICU]	439 nurses [advanced specialist nurse, BSN]
Wieck (2003) *Journal of Nursing Education,* Canada	Nursing science, Nursing education/To explore what students want the faculty, options for increasing the number of nursing graduates.	USA [Large state in southern part] 2000	Descriptive statistics, Delphi technique	Generational cohort/survey, focus group	17 nursing schools and a state‐wide educator conference	49 faculty members and 194 students and nurses
Wros et al. (2004) *Nursing and Health Sciences,* Japan [in English]	Nursing science, Health science between East & West/To describe that part of nursing ethics which is universal and that which is particular to Japan and the USA.	USA/Japan 1993, 1994, 1998	Narrative interpretive phenomenology [USA], ethnography [Japan]	Purposive/focus group, individual interview	Critical care wards in USA and University College in Nursing in Japan	15 advanced specialist nurses [USA], 18 nurse educators [Japan]

### 2.2 Data analysis

The studies were analysed using descriptive data synthesis according to Evans (2002). The studies were read repeatedly by two authors, separately, to gain a sense of the Global Nursing issues presented in the data as a whole. Furthermore, the content of the data was divided by choosing a unit of analysis. In the reading, attention was paid to both the details of accounts and to what each study described. Then, the key components were collected from each study and listed. After this listing of key components, they were also discussed together by the two authors. Furthermore, the process of identifying common areas continued. Differences and similarities were compared during this process. Similar key components were categorized into subcategories. The subcategories in turn constituted five overall explanatory categories (Table 4).

**Table 4:** Subcategories and explanatory categories

Subcategories	Explanatory categories
Facilitating global nursing practice Strengthening global nursing profession	Global Nursing Arena
Creating a supportive environment for global nursing practice Inequalities in creating a supportive environment for global nursing practice Empowering development of global nursing identity Inequalities in development of global nursing identity Enhancing inclusive global nursing ethics	Global Nursing Working Environments
Managing changes in gender, class and generational cohorts Gender and class issues make a difference in various contexts	Global Nursing Workforce Management
Enabling growth and development in global education and learning Inequalities in enabling growth and development in global education and learning Empowering development of professional acknowledgement Inequalities in development of professional acknowledgement Enhancing global competencies Inequalities in enhancing global competencies	Global Nursing Competencies
Political disparities in global networking Supporting leaders in global networking	Global Nursing Networking

## 3 Findings

Global Nursing was first and foremost published in Western journals, mostly represented by the United Kingdom (UK) and the United States (USA). Both educational and practice settings were presented and the participants were nursing students, nurses with varying educational levels and faculty members.

Global Nursing is described by key findings resulted in five categories; Global Nursing Arena, Global Nursing Working Environments, Global Nursing Workforce Management, Global Nursing Competencies and Global Nursing Networking. Overview of the explanatory categories in the included studies is presented in Table 5.

**Table 5:** Overview of the authors and the explanatory categories

Authors	Global Nursing Arena	Global Nursing Working Environments	Global Nursing Workforce Management	Global Nursing Competencies	Global Nursing Networking
Brunetto et al.	X		X		
Garner et al.	X	X		X	X
Gerrish & Griffith	X	X	X		
Gutierrez et al.	X		X	X	
Harrowing et al.	X	X			X
Havens et al.	X	X	X		
Kim et al.	X	X	X	X	X
Lesia & Roets	X	X	X		
Meum et al.	X			X	
Ortiga	X	X		X	X
Squires & Juárez	X	X	X		
Swenson et al.	X				X
Toren et al.	X	X	X		
Tourangeau et al.	X	X		X	
Zhou et al.	X	X	X		
Zinsli & Smythe	X	X	X	X	X
Walton‐Roberts	X	X		X	X
Weng et al.	X	X	X		X
Wieck	X		X	X	
Wros et al.	X	X	X		X

### 3.1 Global Nursing Arena

Different areas have been addressed in the Global Nursing Arena. At the beginning of the millennium, perspectives on nursing education and nursing values in global contexts were topics of concern (Gerrish & Griffith, 2003; Kim, Woith, Otten, & McElmurry, 2006; Swenson, Salmon, Wold, & Sibley, 2005; Wieck, 2003; Wros, Doutrich, & Izumi, 2004). Moving towards the mid‐2010s, global leadership, global competencies and networks have arisen as important questions in the Global Nursing debate (Garner, Metcalfe & Hallyburton, 2009; Harrowing, Mill, Spiers, Kulig, & Kipp, 2010). At the beginning of 2020, the use of nurses’ professional competence in relation to vulnerability in health on a global scale and forming nursing competence in international work has dominated the Global Nursing arena (Brunetto, Farr‐Wharton, & Shacklock, 2012; Gutierrez, Candela, & Carver, 2012; Lesia & Roets, 2013; Squires & Juárez, 2012; Toren, Zelker, & Porat, 2012; Walton‐Roberts, 2012; Zhou, Windsor, Theobald, & Coyer, 2011; Zinsli & Smythe, 2009). Finally, in the mid‐2020s, the question of global leadership, nursing faculty competence, and discussions on nurse professionals and nurse faculty retirement has been key issues (Havens, Warshawsky, & Vasey, 2013; Meum, Ellingsen, Monteiro, Wangensteen, & Igesund, 2013; Tourangeau et al.,^2014^). During recent years, the vulnerability of nurse professionals’ educational and work status has been addressed (Ortiga, 2014; Weng, Huang, Chen, & Chang, 2015).

### 3.2 Global Nursing Working Environments

In the articles, Global Nursing practice in dynamic working environments was influenced by predictors such as encouragement in the work place; an organizational culture that values diversity and an inclusive social atmosphere (Garner et al., 2008; Gerrish & Griffith, 2003; Harrowing et al., 2010; Havens et al., 2013; Kim et al., 2006; Toren et al., 2012; Tourangeau et al., 2014; Weng et al., 2015). Nurse professionals’ equal opportunities for professional practice, use of competence and collaboration in academic and practice contexts were described as essential in supportive environments (Garner et al., 2008; Gerrish & Griffith, 2003; Harrowing et al., 2010; Wros et al., 2004). Creating a supportive environment for Global Nursing practice and research was described to require an appropriate ethical framework based on the actual context (Harrowing et al., 2010; Weng et al., 2015; Wros et al., 2004). Hence, it was noted that the tendency towards paternalism that may accompany research based on Western ethical traditions could be moderated in a milieu of collaboration (Harrowing et al., 2010). However, the power of joint nursing values and ethics was recognized in supportive environments and highlighted by Wros et al. (2004) as in the following citation: “in reviewing the data, it is apparent that nursing values and ethical concerns transcend culture” (p. 134).

Challenges in global practice environments were mentioned and often linked to inequalities in nursing practice such as marginalization and differences in professional equality (Kim et al., 2006; Lesia & Roets, 2013; Squires & Juárez, 2012; Toren et al., 2012; Walton‐Roberts, 2012; Zhou et al., 2011; Zinsli & Smythe, 2009). Many nurse professionals live in countries plagued by social instability and civic unrest, which can hinder growth in nursing identity and development of professional competence (Ortiga, 2014; Walton‐Roberts, 2012; Zinsli & Smythe, 2009). This was explained to lead to the deprofessionalization of nurses and deskilling with limited employment prospects (Walton‐Roberts, 2012; Zhou et al., 2011). Moreover, it was also described that nurse professionals without resources are often stuck in their localities (Squires & Juárez, 2012). For example, medical supply inventory management issues were mentioned as good illustrations of how context matters, as nurses in high‐income countries would not expect to be without medications for more than a few hours or materials for patient care for more than a shift (Squires & Juárez, 2012). Zhou et al. (2011) and Walton‐Roberts (2012) explained how overseas nurses with low status or lack of practice in the field of specialty expressed feelings of social exclusion. Zhou et al. (2011) stated that differences are socially constructed and reveal the mechanisms underlying discrimination and racism.

### 3.3 Global Nursing Workforce Management

Managing changes in gender, class and generational issues were described as essential when facilitating the Global Nursing practice (Brunetto et al., 2012; Havens et al., 2013; Lesia & Roets, 2013; Squires & Juárez, 2012; Toren et al., 2012; Wieck, 2003; Zhou et al., 2011; Zinsli & Smythe, 2009). For the nursing workforce, preferences on work engagement and subordinate communication followed generations (Brunetto et al., 2012; Gerrish & Griffith, 2003; Havens et al., 2013). According to Toren et al., the influence of gender and academic or non‐academic backgrounds were obvious when nursing students were choosing a workplace. Additionally, Wieck (2003) and Weng et al. (2015) explicated that the expectations from nurse professionals included a guarantee of growth in the nursing profession, less hierarchical environments, a determined innovation climate and support to work in various healthcare systems. Weng et al. (2015) also stated that, “Innovation climate fully mediated the relationship between transformational leadership and nurse innovation behaviour. Nurses could perceive the hospital's encouragement and a high degree of support for innovation in their hospitals” (p. 436).

Challenges in management were related to class, gender and power hierarchies, which negatively affected nurses’ perspectives on work (Brunetto et al., 2012; Gerrish & Griffith, 2003; Kim et al., 2006; Tourangeau et al., 2014; Zhou et al., 2011). For example, female nurse migration could perpetuate elements of patriarchy (Wros et al., 2004). Accordingly, nurses’ professional power with respect to diagnostic and prognostic disclosure has been negatively affected by hierarchies.

### 3.4 Global Nursing Competencies

The development of global competencies was explained as one of the great opportunities the nursing profession is facing in the future (Garner et al., 2009; Kim et al., 2006; Wieck, 2003). In the studies, these competencies were closely linked to global leadership. It was also stated that global leaders have knowledge about political operations, politically charged environments and their role in international nursing organizations (Garner et al., 2009; Kim et al., 2006). The view that global competencies could be extended by new educational models, allocated creative projects, and skilled nursing faculty was presented in the studies (Garner et al., 2009; Gutierrez et al., 2012; Kim et al., 2006; Meum et al., 2013; Tourangeau et al., 2014). In addition, education could teach students to apply leadership theories, co‐constructing global standards, collaborative activities in nursing research and an active use of nursing values or nursing ethics (Gutierrez et al., 2012; Meum et al., 2013; Wieck, 2003). Broadened perspectives of global competencies and effective leadership skills were noted by Garner et al. (2009) who stated that “The students gained awareness of Global Nursing issues (advocacy), presented research on topics of interest (activism) and contributed to a continuing education workshop based on their experiences (professional accountability)” (p. 105).

In the studies, there were descriptions of challenges with regard to global competencies (Ortiga, 2014; Walton‐Roberts, 2012; Zinsli & Smythe, 2009). This could, for example, be lack of equal recognition of knowledge in the field of nursing, inability to demonstrate nurse autonomy and negative connotations ascribed to nurses’ professional competence (Ortiga, 2014; Zinsli & Smythe, 2009). Challenges in terms of inequalities were further described by Ortiga (2014), when nurse educators outside the Western world are forced to negotiate an overloaded curriculum, due to the influx of aspiring migrants into nursing programmes. Differences were also indicated for international disaster and emergency nurses, when their growth in global competence was diminished due to personal danger and overwhelming needs (Zinsli & Smythe, 2009).

### 3.5 Global Nursing Networking

Global strategies were explained to build stronger international bodies (Kim et al., 2006; Swenson et al., 2005). Garner et al. (2009) also stated that global networking is promoted by global strategies and could be further developed in joint creative projects. In these projects, it was important to discuss activities for global networking (Garner et al., 2009; Kim et al., 2006; Swenson et al., 2005). It was also stated that the understanding of the impact of politics on healthcare provision and how it affects global networking could broaden the global perspectives (Garner et al., 2009; Harrowing et al., 2010; Ortiga, 2014; Walton‐Roberts, 2012; Zinsli & Smythe, 2009). Furthermore, growth in leadership competencies and an increased sense of both professional and personal nursing community was described as essential (Garner et al., 2009; Kim et al., 2006; Weng et al., 2015).

In the studies, it was stated that political differences in global networking are challenging. In some Global Nursing contexts, political disparities still exist, for example, international nurse migration has not been addressed (Ortiga, 2014; Walton‐Roberts, 2012; Wros et al., 2004; Zinsli & Smythe, 2009). According to Ortiga (2014), many countries bear the burden of nursing employability and nations lower down the chain supply labour to wealthier countries, “Nurse educators expressed wanting their students to be resourceful and quick on their feet, given the lack of proper facilities in many local hospitals. Yet, as schools became focused on producing nurses for export, the educators faced the dilemma of teaching students how to practice ‘first world’ nursing in a ‘third world’ context” (p. 67).

## 4 Discussion

The aim of this study was to describe key findings of Global Nursing in empirical nursing studies. The findings are described in five categories. The Global Nursing Arena category showed that the focus has shifted from an interest in education and values to a focus on how a nursing faculty can adopt sustainable strategies to engage in Global Nursing leadership (cf. Garner, et al., 2008). Therefore, we suggest that education for nurses has to contain broadened fundamental knowledge and awareness of dominant areas such as economics, demographics, politics and social constitutions. Our interpretation is that technological developments and policy changes during recent decades have increased the meaning and implication of Global Nursing in both nursing education and practice. The debate has matured considerably and become more extensive over time.

In the Global Nursing Working Environments category, discrepancies in work environments due to globalization might transform evidence‐based nursing practice or research to support inequalities in the global village (cf. Harrowing et al., 2010). Our concern is that global care chains demonstrate the south to north migration of nurses and this global movement reinforces inequities. Migration is structured by the power hierarchies embodied in class, ethnicity, gender, nationality and race especially in the era of Global Nursing shortage (cf. England, 2015; Xu, 2015). Widding Isaksen (2012) described how the Norwegian welfare state is becoming a global employer and global nurse recruitment generates transnational spaces of care. Furthermore, international nurse recruitment is not a win–win situation and the idealized image of social justice and gender equality needs to be critically examined. We suggest that power hierarchies and global justice or fairness as a deeper human right need to be discussed in practical contexts in nursing education. By training nurses to discover stereotypical behaviour, hidden prejudices and normative structures, nursing education has the opportunity to explain how to counteract inequities. Consequently, it is important that the use of suitable solutions follows local contexts in nursing practice and research.

The consequences of power hierarchies in healthcare systems cause differences in nursing, a loss of identity for nurses and a risk for deskilling in the profession (cf. Toren et al., 2012; Widding Isaksen, 2012). Gender and class are often constituted as “otherness”. Moreover, identity such as the Self is relative and changing compared with the Other (Eriksson‐Baaz, 2005). Said (1978) argued that Western societies have used their hegemonic position of interpretation to antagonize those who are non‐Western. Throughout colonial history, there have been descriptions of qualities such as the Other; to be childish, irrational and depraved in contrast to the Self which has been ascribed with qualities as rational, mature, virtues and normal (Eriksson‐Baaz, 2005; Said, 1978). White Western culture, historically and still today, has monopolized science, knowledge, clinical practice and health‐promoting models. Anderson et al. (2009) claimed that it is our lack of concern about the social disadvantage of “others” at local, national and global levels that leads to serious health disparities. Furthermore, social justice is highlighted as being of importance for nurses. Our interpretation is that these cases have to be further investigated. Education can prevent inequalities by training nurses to make conscious choices in nursing activities when power hierarchies occur in healthcare systems.

The Global Nursing Workforce Management category highlighted transformational leadership, growth in the nursing profession, nurse innovation behaviour and high mobility. However, nurse migration and the export of nurses create business and profits for many countries, organizations and agencies (cf. Zhou et al., 2011). This raises the question of how inequality in nurse migration has become a moral problem. England (2015) concluded that analyses of linkages between globalization, migration and care are essential. In countries with nurse export, there is a lack of healthcare professionals, which in turn results in a lack of health care, an economic imbalance and vulnerability among people (cf. Kaelin, 2011). Accordingly, in developing countries, nurse recruitment hinders people in their ability to make use of their political liberties (ibid.). Thus, The World Health Organization calls for principles of transparency, fairness and the promotion of sustainability of health systems in developing countries (2010). Our interpretation is that nursing education and practice are challenged in meeting the expectations of nurse professionals and in guaranteeing growth in the nursing profession.

Global Nursing Competencies have shifted in focus to engagement in a global leadership and professional activism (cf. Garner et al., 2009). Based on the findings, we stress that nursing education provides a great opportunity to promote global competencies and explain diminishing aspects important for the nursing profession in nursing practice. Furthermore, a mission is to prepare students to train global awareness by actions and reflection on their experiences. According to our findings, we recommend an ethical framework and nursing actions as useful tools in the efforts to reduce inequities and inequalities in Global Nursing practice (cf. Grootjans & Newman, 2013; Gutierrez et al., 2012; Meum et al., 2013; Wieck, 2003).

Global Nursing Networking explained strategies that could be helpful and used to further develop joint creative projects (cf. Garner et al., 2009; Kim et al., 2006; Swenson et al., 2005). Moreover, global leadership was described as being of vital importance. One solution for change in nursing challenges is proposed by Nardi and Gyurko (2013). They highlight that Global Networking can be used to design new education models that suit global healthcare needs, pooling teaching resources, designing and using databases across organizations to track and project faculty needs. Accordingly, there is a need for innovation in nursing practice to accommodate the huge challenges facing the future of nursing (ibid.). We claim that work in different healthcare systems and team building together with a long‐term innovation climate is important for Global Nursing Networking. In both nursing education and practice, there are great opportunities for nurse professionals and faculty to collaborate in the global community.

Finally, we discuss why we only found two studies that were published outside the Western world although Global Nursing exists in the entire world. Furthermore, the results showed that the UK and the USA are active participants writing about Global Nursing issues, which means that it is essentially a Western perspective that is being shown. This is a question of concern as the concept of a global representation might also be connected to values that have their origin in colonialism. We stress the importance of postcolonial awareness in nursing when dealing with matters of globalization. Postcolonialism has been described as the response to the marginalization of Western cultures’ values and norms (cf. Said, 1978; Shakib, 2011). This is a concern as the Western context needs to undergo a series of changes to tackle its colonial past even in nursing. We would like to add that this is a critical aspect in advocating for a global agenda in nursing.

### 4.1 Methodological considerations

The data collection and data analysis in this review will be critically discussed based on various parts linked to *trustworthiness* (cp. Sandelowski, 1993; Rolfe, 2006).

*Credibility* involves aspects such as how to source articles, how to select key components and how well the explanatory categories cover the data. Careful considerations have been taken when planning and working with the appraisal of each phase in the review process.

The appraisal was based on inclusion and exclusion criteria in the search strategy. Subjective reading and evaluation with the modified checklist with evaluation sections related to Kmet et al. (2004). For the studies with qualitative methods, the appraisal process for each study included has been taken into account through an investigation of the reflexivity of the researcher, the participants and the description of context. For the surveys included, the appraisal process has been taken into account through an investigation of validity and reliability of sample, data collection, analysis methods, setting and participants, to enhance the understanding of the findings in this evaluation.

*Transferability* refers to the extent to which findings can be transferred to other settings. The number of organizations taking part in the study, where they are based and the number of participants involved have carefully been described to get sufficient contextual information about the studies included. Accordingly, the data collection methods that were employed and the time period over which the data were collected, has been reported. However, it is up to the reader to decide whether it is possible to transfer outcomes to other contexts.

*Dependability* of the data analysis was ensured by showing the procedure of coding and formulating the explanatory categories according to Evans (2002). In this review, the two authors who conducted the analysis reflected on and discussed the content and categorization models. To address the dependability issue, the processes in the study have been reported in detail and include the researcher design and how it was carried out. For this review, only the term Global Nursing in the field of nursing was accepted in the search and research literature. Other related terms were excluded due to the large amount of material and the intention to obtain a clear description with respect to the Global Nursing phenomenon. In addition, some research articles had to be excluded due to non‐availability or translation problems from the original language into English. Consequently, these aforementioned issues might limit the search and cause selection bias. *Confirmability* is the investigator's comparable concern to objectivity. In this review, a detailed methodological description allows the reader to determine how far the data and constructs emerging from it may be accepted.

## 5 Conclusion

This literature review emphasized that nursing education provides a great opportunity to promote global relationships through global networking. Global Nursing offers wider awareness in nursing knowledge and contributes to a nursing profession equipped for today's challenges and global work with inequalities.

## Conflict of Interest

The authors declare no conflict of interest or external support in the form of grants.

## Author contributions

MK, AK, AMRH, HE: Study design, data collection, data analysis and manuscript preparation.

All the Authors have agreed on the final version and meet at least one of the following criteria [recommended by the ICMJE (http://www.icmje.org/ethical_1author.html)]:

- substantial contribution to conception and design, acquisition of data or analysis and interpretation of data;
- drafting the article or revising it critically for important intellectual content.
